# Uncovering pathology, subjective cognitive complaints, and sex in early Alzheimer's disease

**DOI:** 10.1177/13872877251393268

**Published:** 2025-11-11

**Authors:** Sophie Boutin, Bérengère Houzé, Alexa Pichet Binette, Simona Maria Brambati, for the Alzheimer's Disease Neuroimaging Initiative

**Affiliations:** 1 Department of Psychology, Université de Montréal, Montréal, Québec, Canada; 2 Centre de Recherche de l’Institut Universitaire de Gériatrie de Montréal, Montréal, Québec, Canada; 3 Department of Pharmacology and Physiology, Université de Montréal, Montréal, Québec, Canada

**Keywords:** Alzheimer's disease, amyloid, cognitive dysfunction, sex characteristics

## Abstract

**Background:**

Subjective cognitive complaints may correlate with cerebral amyloid-β (Aβ) levels in the early phases of Alzheimer's disease (AD). The relationship between sex, AD pathology, and complaints remains unclear.

**Objective:**

Our study aims to (1) explore the relationship between Aβ pathology, assessed with two complementary measures, and subjective cognitive complaints across multiple domains in cognitively unimpaired (CU) individuals and those with mild cognitive impairment (MCI); (2) assess which subjective cognitive complaints can differentiate Aβ-positive from Aβ-negative individuals, CU from MCI, and progressors from non-progressors; and (3) evaluate sex differences in these relationships.

**Methods:**

In 418 CU older adults and 408 with MCI from the ADNI cohort, we examined associations between Aβ, subjective cognitive complaints and sex, controlling for age, education, depression, and anxiety.

**Results:**

In CU individuals, higher Aβ levels correlated with more severe language and visuospatial complaints. MCI individuals with elevated Aβ reported more severe memory, language, and planning complaints. Memory, language, planning, and organization complaints predicted risk of MCI and clinical progression. Sex differences emerged in the association between Aβ and visuospatial complaints, and in complaint types predicting Aβ positivity and cognitive impairment.

**Conclusions:**

Subjective cognitive complaints in memory and non-memory domains (language, visuospatial, and executive functions) may signal cognitive decline risk due to their association with AD biomarkers and clinical progression. Sex differences highlight the need for personalized approaches in AD early diagnosis and disease progression monitoring.

## Introduction

With new interventions emerging to slow the progression of Alzheimer's disease (AD), it is crucial to focus research on early AD phases to target personalized medicine before the first neurocognitive deficits. At the preclinical phase of AD, individuals are cognitively unimpaired (CU), but at higher risk of cognitive decline because of positive AD biomarkers such as amyloid-β (Aβ) proteins that start to accumulate decades before the first cognitive symptoms of the disease.^1–4^ At the stage of mild cognitive impairment (MCI), individuals show cognitive deficits that do not yet lead to functional impairment.^5^ In both of these early phases, individuals might also report subjective cognitive complaints, or subjectively experienced worsening of cognitive capacities,^6^ at varying severity level. Although cognitive complaints are frequently reported among older adults aged 60 and above,^7^ previous studies showed that more severe subjective cognitive complaints predict future AD-related cognitive deficits.^8–11^ Nonetheless, significant uncertainties remain about how to define, measure, and understand the practical importance of subjective cognitive complaints in the early AD stages (CU and MCI).

Although previous studies have investigated the relationship between subjective cognitive complaints and AD biomarkers, such as Aβ proteins, they often focus on specific aspects of this association in isolation, without providing a comprehensive overview of the relationship. As such, some studies focus primarily on memory complaints,^12,13^ others use a single global question to assess complaints severity^14,15^ or rely on one measure of Aβ (e.g., continuous levels or binary Aβ status),^16,17^ while others restrict their analyses to a specific clinical group (e.g., CU, subjective cognitive decline or MCI)^18–22^ or to a cross-sectional design. As a result, specific but fragmentary gaps remain in understanding how Aβ pathology relates to the severity of domain-specific cognitive complaints beyond the memory domain across the AD continuum.

First, a recent meta-analysis highlighted that the strength of associations between AD biomarkers and subjective cognitive complaints can vary depending on the questionnaire used and the cognitive domain assessed.^23^ A domain-specific approach to assess the severity of complaints is also clinically relevant because early AD clinical presentation is heterogeneous: in addition to memory, complaints in domains such as language (e.g., word-finding) and executive functions have also been linked to increased risk of AD-related decline,^24,25^ yet remain underexplored.^16,18^ Second, combining continuous and binary (Aβ+/Aβ−) measures of Aβ provides complementary insights into pathology throughout the disease continuum (CU and MCI).^1,^^26–28^ Third, although prior studies have linked subjective cognitive complaints to Aβ burden in both CU^20,21,29^ and MCI^22,30^ individuals, few have examined how this relationship evolves from preclinical to prodromal AD within the same cohort, while also using longitudinal data to assess the role of complaints in clinical progression. This has clear clinical value, as it may inform stage-specific screening strategies and clarify which types of complaints are most informative at different points along the disease continuum. By combining these elements in a large, well-characterized longitudinal cohort, our study uniquely contributes to the field by offering a more comprehensive understanding of how subjective cognitive complaints across domains relate to Aβ pathology, highlighting their potential utility for early detection and tracking clinical progression across disease stages.

Another aspect that is often neglected in this literature is the impact of sex on subjective cognitive complaints severity across domains and their relationship with Aβ pathology. This is crucial as two thirds of AD dementia patients are women,^31^ even after controlling for women's longer lifespan. Recent studies suggest women face higher risks of AD pathology and cognitive decline,^32–38^ showing more rapid cognitive deterioration^12,15,^^39–49^ and greater pathology progression^13,30,43,44,^^50–58^ as Aβ levels increase, yet sex has often been treated as a covariate rather than a variable of interest. One possible explanation for this clinical pattern is the theory of cognitive resilience,^59^ which suggests that women, despite exhibiting higher Aβ levels, tend to maintain superior verbal memory performance in early disease stages.^30,44,56,60^ This resilience, potentially supported by protective factors such as estrogen,^61^ may delay symptom recognition and diagnosis in women.^59,62^ However, as pathology advances, this advantage appears to diminish, and women may experience faster and more pronounced cognitive decline.^12,47,48^ These findings highlight a limitation of relying solely on objective cognitive tests to study sex differences in early AD, as ceiling effects may mask subtle cognitive changes in these populations. In this context, subjective cognitive complaints may offer a more sensitive proxy for detecting sex-specific differences in early disease stages. Despite its relevance, studies examining sex differences in subjective cognitive complaints severity have focused mainly on the subjective cognitive decline stage, a supposed intermediate AD stage between normal cognition and MCI and provided contradicting results. While most highlighted women reported more severe memory complaints, some suggested either men reported more severe complaints or no sex differences.^63–67^ Thus, research on the complex relationship between sex, subjective cognitive complaints and Aβ pathology in the early AD stages remains needed. This has meaningful implications for developing sex-sensitive screening tools that account for biological and experiential differences between women and men.

To address these gaps within a single design, the study pursues three main objectives. First, this study aims to explore the relationship between Aβ pathology (using both continuous Aβ levels and Aβ+/Aβ− categorization), and cognitive complaints severity across domains in CU and MCI individuals. We hypothesize that higher Aβ pathology, especially when using the more sensitive continuous measure as opposed to the binary status, will be associated with greater severity of subjective cognitive complaints, particularly in the memory, language, and executive function domains, in line with previous findings. Second, we aim to identify which subjective cognitive complaints predict cerebral Aβ status (Aβ+/Aβ−) and cognitive status (CU or MCI). As a sub-objective, we also examine which specific complaints are associated with clinical progression (progressing from CU to MCI or from MCI to AD). We further hypothesize that the severity of complaints in the above-mentioned key cognitive domains will predict Aβ positivity, as well as a greater likelihood of MCI and clinical progression, reflecting early markers of AD-related decline. Our third objective focuses on sex differences in early AD by investigating: (a) whether Aβ pathology association with subjective cognitive complaints severity varies by sex, (b) sex-specific differences in complaints severity across domains, and (c) whether the most predictive cognitive complaints for Aβ status and cognitive status differ between men and women. Given the limited and sometimes conflicting findings on sex differences in subjective cognitive complaints, we based our hypotheses on the literature examining sex differences in objective cognitive performance. Considering women's often superior verbal memory abilities in early AD, we hypothesize that: (a) the association between Aβ and complaints severity may be weaker in CU women due to cognitive resilience, potentially strengthening at the MCI stage; (b) CU women may report less severe memory and language complaints, with a reversal in MCI as their cognitive advantage diminishes; and (c) memory and language complaints may be less predictive of Aβ positivity and cognitive status in CU women compared to men or women with MCI.

## Methods

Data used in the preparation of this article were obtained from the Alzheimer's Disease Neuroimaging Initiative (ADNI) database (adni.loni.usc.edu). The ADNI was launched in 2003 as a public-private partnership, led by Principal Investigator Michael W. Weiner, MD. The primary goal of ADNI has been to test whether serial magnetic resonance imaging, positron emission tomography (PET), other biological markers, and clinical and neuropsychological assessment can be combined to measure the progression of MCI and early AD. For up-to-date information, see www.adni-info.org.

### Participants

For our study, we selected only CU and MCI individuals from the ADNI database. In the general ADNI inclusion criteria, participants are considered CU if they present: 1) a Mini-Mental State Examination (MMSE) score between 24 and 30; 2) a Clinical Dementia Rating score of 0; 3) above education-adjusted scores on the delayed Paragraph Recall task from the Wechsler Memory Scale Logical Memory II and 4) no significant impairment in cognitive functions or activities of daily living. To be included in the MCI group, participants must present: (1) memory complaints that are verified by their study partner; (2) a MMSE score between 24 and 30; (3) a Clinical Dementia Rating score of 0.5; (4) below education-adjusted scores on the delayed Paragraph Recall task from the Wechsler Memory Scale Logical Memory II and (5) sufficiently preserved cognitive functions and functional performance such that AD diagnosis cannot be made. Study-specific inclusion criteria include (1) having English as primary language to have a more homogeneous sample (2) being 65 years old or older (to be in line with older adults’ literature) (3) having available self-rated Everyday Cognition (E-Cog) and (4) Aβ PET biomarkers data. The ADNI protocols were approved by all the Institutional Review Boards of the participating institutions. All participants provided written informed consent prior to enrolment in the study. For full description of Institutional Review Boards procedures and approvals, see: https://adni.loni.usc.edu/help-faqs/adni-documentation/. In addition to ADNI ethical considerations, this study was also approved by the Education and Psychology Research Ethics Committee of *Université de Montréal* (CEREP) and by the Aging-Neuroimaging Research Ethics Committee of the *Centre* *Intégré Universitaire de Santé et de Services Sociaux du Centre-Sud-de-l’Île-de-Montréal* (approval no. CER VN 23-24-27) on December 7, 2023 (renewed on January 13, 2025).

### Subjective cognitive complaints and other clinical assessments

The severity of subjective cognitive complaints was measured using the self-reported E-Cog questionnaire. The E-Cog is a validated 39-item questionnaire assessing an individual's subjective complaints within six cognitive domains: memory, language, visuospatial, planning, organization and divided attention. Response to each item ranges from 1 to 4 (1 = no change or performs better than 10 years ago; 2 = occasionally performs the task worse than 10 years ago but not all of the time; 3 = consistently performs the task a little worse than 10 years ago; 4 = performs the task much worse than 10 years ago). Participants could also answer 9 = I don’t know, but these answers were treated as missing values in the present study.

Considering the possible impact of depression and anxiety symptoms on the subjective experience of cognitive changes,^68^ the results at the Geriatric Depression Scale^69^ and Neuropsychiatric Inventory^70^ (anxiety subscore) were extracted to include as covariates in our analyses alongside age and years of education. MMSE scores, *APOE* ε4 allele carriership and race/ethnicity were also extracted to better characterize our sample.

### Biomarkers

Aβ pathology was measured both as the actual quantity and using the Aβ+/Aβ− status based on established thresholds. To assess global Aβ quantity in the whole brain, we used the standardized uptake value ratio (SUVR) obtained with PET imaging (Florbetapir (F18 AV-45) tracer). The Aβ SUVR available in ADNI was calculated in a cortical summary region that includes frontal, anterior/posterior cingulate, lateral parietal and lateral temporal regions using the whole cerebellum as the reference region. The same SUVR value was used to classify individuals who were at higher risk of cognitive decline, where individuals who have an Aβ SUVR value of more than 1.11 are considered Aβ+ and those who have an Aβ SUVR value below this cutoff are considered Aβ−. The complete descriptions of the collection, transportation, and analyses protocols are provided in the ADNI procedural manual at www.adni-info.org.

Since clinical, cognitive, and imaging tests were not necessarily done at the same time point in ADNI, we only kept the data with less than 6 months between the time of diagnosis (CU or MCI) and the dates of PET scans and clinical assessments.

### Statistical analyses

All statistical analyses were performed using R version 4.2.2.

To characterize CU and MCI groups, one-way ANOVAs were performed to determine group differences on demographics, according to Aβ levels and sex.

To address the first objective of the study, we ran two analyses. First, to examine the association between Aβ levels (as continuous values) and the severity of subjective cognitive complaints, we used general linear models separately in CU and MCI individuals. In every model, we used the global Aβ quantity as the independent variable and either the overall E-Cog score (sum of all 39 items) or the subscores of the six cognitive domains (mean of eight memory items; nine language items; seven visuospatial items; five planning items; six organization items; and four divided attention items, requiring that at least 75% of items were answered to calculate the subscores) as the dependent variables. Then, to examine the link between Aβ pathology and the severity of subjective cognitive complaints using rather the Aβ status measure, we performed separate two-way ANOVAs on the 39 E-Cog item scores, testing for the main effects of Aβ status (Aβ+/Aβ−) and sex as well as their interaction on the severity of subjective cognitive complaints. The decision to use parametric statistical tests instead of nonparametric statistical tests to analyze E-Cog ordinal data with low variability is justified by the relatively high number of participants per group which allows us to treat ordinal data as continuous. This approach was also adopted in previously published work with similar data.^18^

To address the second objective of the study, which is to assess which subjective cognitive complaints severity across domains can predict cerebral Aβ status and cognitive status (CU or MCI), we performed logistic regressions. Separate models were used for the CU and MCI groups to determine whether and which of the 39 E-Cog item scores could predict Aβ status (Aβ+ or Aβ−), and another model was used with the whole sample (CU and MCI combined) to determine the predictors of their cognitive status (CU or MCI). We additionally performed a complementary analysis to identify which complaints distinguished progressors (individuals who progressed from CU to MCI [n = 35] or MCI to AD [n = 63]) from non-progressors (stable CU [n = 400] or MCI [n = 326]). Separate logistic regressions were conducted for CU and MCI groups, including all participants with at least one follow-up diagnosis within 5 years of baseline. The 39 baseline E-Cog item scores were entered as predictors in the models.

To address our third objective, which is to examine the role of sex, we first included an interaction term between the E-Cog score and sex in every general linear model that we ran for investigating the first objective. This allows determining whether the association between Aβ pathology and the severity of subjective cognitive complaints differs by sex. We also conducted sex-stratified analyses by performing the linear models separately in men and women (see Supplemental Material). Then, by including the main effect of sex and its interaction with Aβ status in the ANOVA models used for investigating the first objective, we can determine whether there are sex differences in complaints severity across domains and whether the effect of sex on complaints severity differs depending on Aβ status. Finally, to determine whether the predictors of Aβ status (Aβ+ or Aβ−) and cognitive status (CU or MCI) differ according to sex, we performed the logistic regression models used for investigating the second objective separately in men and women.

Age, years of education, depression and anxiety symptoms were included as covariates in all statistical models, and p-values were corrected using the less conservative Benjamini & Hochberg method for multiple comparisons.^71^

## Results

### Description of participants

In total, 418 CU and 408 MCI individuals were included in the present study (Tables 1 and 2). Six participants (two CU and four MCI) were excluded because they had not answered at least 11 questions on the E-Cog and two participants (one CU and one MCI) were excluded because they had not PET data available. The CU sample was 44.3% male and 55.7% female, the mean age was 75.4 years and participants were highly educated (16.7 years of education on average). The mean MMSE score for the whole CU sample was 29.0 and the mean Aβ SUVR value was 1.13. To allow better comparison with other studies in the field, the Aβ SUVR value was transformed in centiloid, an increasingly used Aβ metric, and was added in the tables. Two-sample t-tests showed that, without considering their Aβ status, women had significantly higher Aβ levels [t(414) = −3.31, *p* *=* 0*.*001], higher performance in memory tasks [t(406) = −6.74, *p* < 0.001] and lower performance in visuospatial tasks [t(401) = 2.64, *p* *=* 0*.*009] compared to men. The MCI sample was 60.8% male and 39.2% female, the mean age was 76.4 years and participants had 16.1 years of education on average. The mean MMSE score for the whole MCI sample was 27.8 and the mean Aβ SUVR value was 1.23. However, unlike the CU sample, there was not a significant sex difference in Aβ levels [t(338) = 0.45, *p* *=* 0*.*653] nor in visuospatial tasks performance [t(351) = −0.60, *p* *=* 0*.*551], although MCI women performed better than MCI men in memory tasks [t(303) = −2.15, *p* *=* 0*.*032]. See Supplemental Material for E-Cog mean subscores and standard deviations per group.

**Table 1.** Demographic and psychological characteristics of CU individuals.

Characteristics	Men_Aβ− (n = 130)	Men_Aβ+ (n = 55)	Women_Aβ− (n = 130)	Women_Aβ+ (n = 103)	F (3,414) and *p* values	Pair-wise comparisons
Age	75.6 ± 6.7	79.0 ± 6.4	73.6 ± 6.1	75.5 ± 5.9	F = 9.69, *p* < 0.001	Men_Aβ+ > Women_Aβ−*** Men_Aβ+ > Women_Aβ+** Men_Aβ+ > Men_Aβ−**
Education	17.1 ± 2.5	17.4 ± 2.3	16.4 ± 2.6	16.1 ± 2.6	F = 5.22, *p* *=* 0*.*002	Men_Aβ− > Women_Aβ+* Men_Aβ+ > Women_Aβ+* Men_Aβ+ > Women_Aβ−*
GDS score (max = 15)	1.02 ± 1.33	0.87 ± 1.25	1.17 ± 1.53	1.16 ± 1.31	F = 0.77, *p* *=* 0*.*512	
NPI (Anxiety) score (max = 12)	0.15 ± 0.79	0.13 ± 0.67	0.04 ± 0.32	0.12 ± 0.55	F = 0.86, *p* *=* 0*.*461	
MMSE score (max = 30)	29.0 ± 1.2	28.7 ± 1.2	29.2 ± 1.2	28.8 ± 1.5	F = 3.28, *p* *=* 0*.*021	Women_Aβ− > Men_Aβ+*
Aβ SUVR (CL)	1.01 ± 0.05 (0.94)	1.30 ± 0.16 (55.53)	1.02 ± 0.05 (2.82)	1.33 ± 0.18 (61.17)	F = 227.70, *p* < 0.001	Men_Aβ+ > Men_Aβ−*** Men_Aβ+ > Women_Aβ−*** Women_Aβ+ > Women_Aβ−*** Women_Aβ+ > Men_Aβ−***
*APOE* ε4 (n/%)	22/16.9	22/40	31/23.8	49/47.6		
White (n/%)	113/86.9	49/89.1	114/87.7	89/86.4		

For numerical variables, we indicated the mean ± standard deviation. The F and p values are the results of group comparisons (ANOVAs). Tukey's HSD test was used as a post hoc test: *p < 0.05, **p < 0.01, ***p < 0.001. The equation used for centiloid transformation is as follows: CL = 188.22×SUVR-189.16.^70^

Aβ−: amyloid levels below threshold; Aβ+: amyloid levels above threshold; *APOE* ε4: at least one ε4 allele on the *APOE* gene; CL: Centiloid; CU: cognitively unimpaired; GDS: Geriatric Depression Scale; MMSE: Mini-Mental State Examination; NPI: Neuropsychiatric Inventory.

**Table 2.** Demographic and psychological characteristics of MCI individuals.

Characteristics	Men_Aβ− (n = 98)	Men_Aβ+ (n = 150)	Women_Aβ− (n = 73)	Women_Aβ+ (n = 87)	F (3,404) and *p* values	Pair-wise comparisons
Age	76.1 ± 6.6	77.2 ± 6.0	76.1 ± 7.3	75.7 ± 6.3	F = 1.22, *p* *=* 0*.*301	
Education	16.8 ± 2.4	16.4 ± 2.9	15.7 ± 2.5	15.3 ± 2.6	F = 5.91, *p* < 0.001	Men_Aβ− > Women_Aβ−* Men_Aβ− > Women_Aβ+*** Men_Aβ+ > Women_Aβ+*
GDS score (max = 15)	1.43 ± 1.40	1.74 ± 1.59	1.99 ± 2.16	2.00 ± 1.75	F = 2.26, *p* *=* 0*.*081	
NPI (Anxiety) score (max = 12)	0.45 ± 1.51	0.55 ± 1.55	0.19 ± 0.72	0.41 ± 1.49	F = 1.04, *p* *=* 0*.*374	
MMSE score (max = 30)	28.4 ± 1.7	27.2 ± 2.1	28.4 ± 1.7	27.4 ± 2.1	F = 11.50, *p* < 0.001	Men_Aβ− > Women_Aβ+** Men_Aβ− > Men_Aβ+*** Women_Aβ− > Women_Aβ+* Women_Aβ− > Men_Aβ+***
Aβ SUVR (CL)	0.99 ± 0.06 (−2.82)	1.39 ± 0.17 (72.47)	1.01 ± 0.05 (0.94)	1.40 ± 0.19 (74.35)	F = 273.60, *p* < 0.001	Men_Aβ+ > Men_Aβ−*** Men_Aβ+ > Women_Aβ−*** Women_Aβ+ > Women_Aβ−*** Women_Aβ+ > Men_Aβ−***
*APOE* ε4 (n/%)	19/19.4	96/64.0	6/8.2	52/59.8		
White (n/%)	95/96.9	144/96.0	67/91.8	79/90.8		

For numerical variables, we indicated the mean ± standard deviation. The F and p values are the results of group comparisons (ANOVAs). Tukey's HSD test was used as a post hoc test: *p < 0.05, **p < 0.01, ***p < 0.001. The equation used for centiloid transformation is as follows: CL = 188.22xSUVR-189.16.^70^

Aβ−: amyloid levels below threshold; Aβ+: amyloid levels above threshold; *APOE* ε4: at least one ε4 allele on the *APOE* gene; CL: Centiloid; GDS: Geriatric Depression Scale; MCI: mild cognitive impairment; MMSE: Mini-Mental State Examination; NPI: Neuropsychiatric Inventory

### Objective 1: relationship between Aβ pathology and the severity of subjective cognitive complaints in CU and MCI individuals

**Figure 1.**
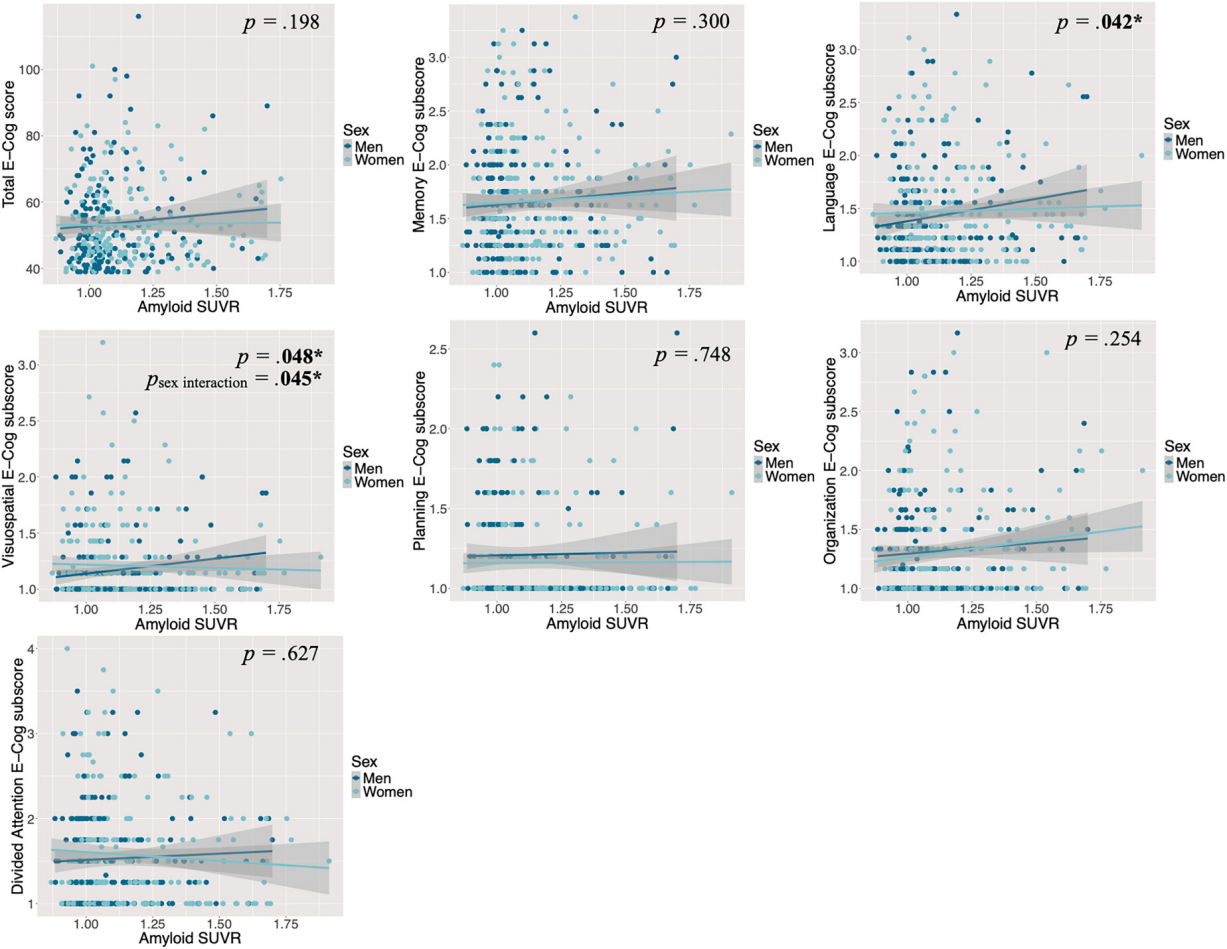
The association between Aβ levels and the severity of cognitive complaints, by sex and domain, controlling for age, years of education and symptoms of depression and anxiety in CU individuals. Reported *p *values reflect the associations between Aβ levels and E-Cog scores for the whole CU sample (men and women combined). Here, higher amyloid-β levels were associated with higher E-Cog scores (more severe subjective cognitive complaints) only in the language and visuospatial domains in CU individuals. In the visuospatial domain, this association is stronger for men (dark blue) than for women (light blue). For the sex interaction, only significant *p*values are shown. CU: Cognitively Unimpaired; E-Cog: Everyday Cognition Questionnaire; SUVR: Standardized Uptake Value Ratio.

**Figure 2.**
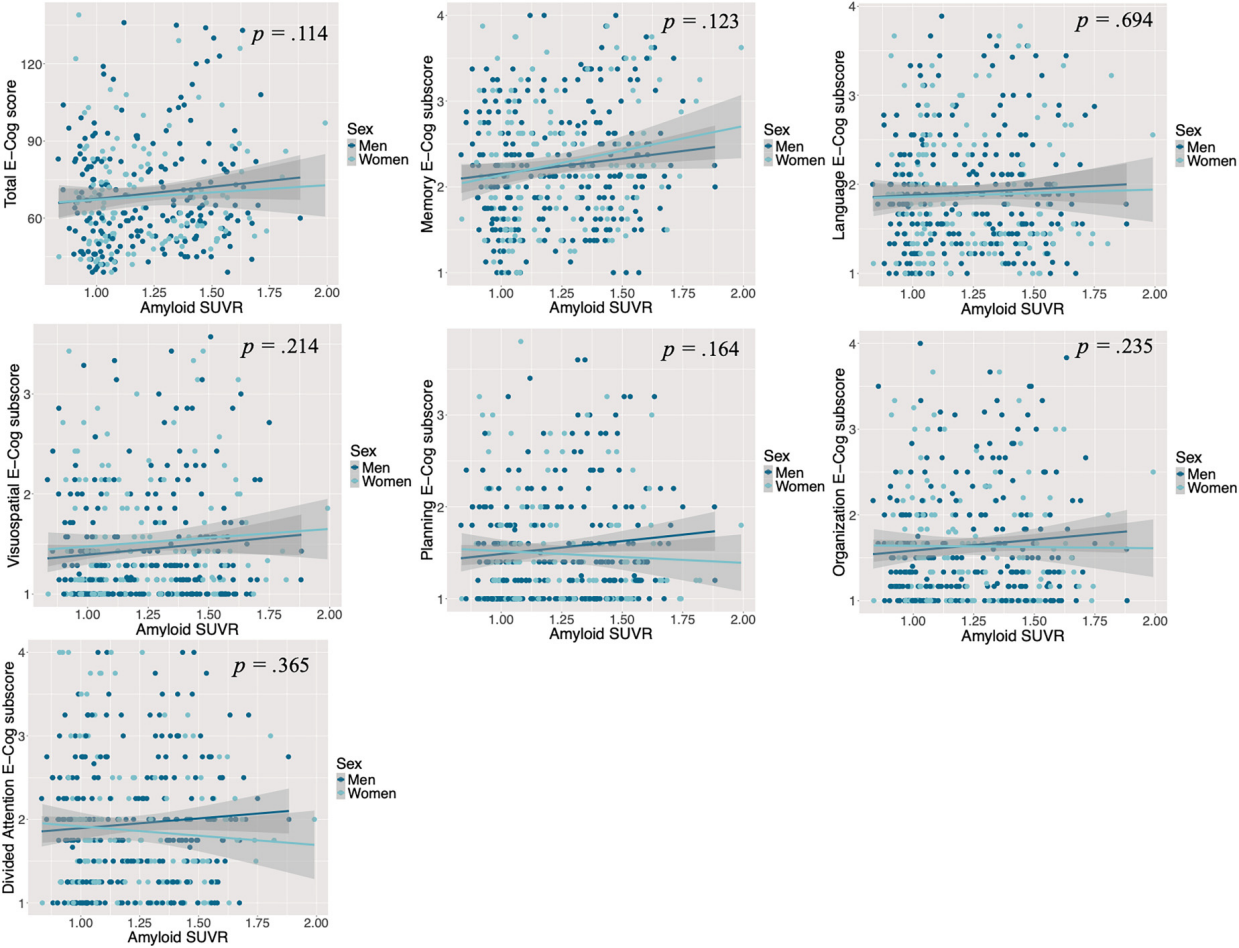
The association between Aβ levels and the severity of cognitive complaints, by sex and domain, controlling for age, years of education and symptoms of depression and anxiety in individuals at the MCI stage. Reported *p*values reflect the associations between Aβ levels and E-Cog scores for the whole MCI sample (men and women combined). Here, Aβ levels were not associated with E-Cog scores in any cognitive domain in MCI individuals. As no significant sex interaction was detected, corresponding *p*values are not presented. E-Cog: Everyday Cognition Questionnaire; MCI: Mild Cognitive Impairment; SUVR: Standardized Uptake Value Ratio.

When we used Aβ as a continuous variable, the results of the general linear models in CU individuals indicated that higher Aβ levels were associated with more severe subjective cognitive complaints exclusively in the language and visuospatial domains (Figure 1), after controlling for age, years of education, and depression and anxiety symptoms. Depressive symptoms, as a covariate, were also found to be associated with increased severity of cognitive complaints, both in the overall assessment and when examining each cognitive domain individually (total E-Cog: t = 5.95, *p* < 0.001; memory domain: t = 6.88, *p* < 0.001; language domain: t = 6.90, *p* < 0.001; visuospatial domain: t = 5.28, *p* < 0.001; planning domain: t = 7.19, *p* < 0.001; organization domain: t = 6.74, *p* < 0.001; divided attention domain: t = 6.35, *p* < 0.001). However, in MCI individuals, the results showed that there was no association between Aβ levels, as measured as the actual quantity, and the severity of subjective cognitive complaints neither in the total E-Cog score nor the 6 cognitive domains (Figure 2), after controlling for covariates. However, like the CU sample, depressive symptoms, as a covariate, were associated with more severe complaints both in the overall assessment and when examining each cognitive domain individually (total E-Cog: t = 6.99, *p* < 0.001; memory domain: t = 7.03, *p* < 0.001; language domain: t = 7.30, *p* < 0.001; visuospatial domain: t = 7.31, *p* < 0.001; planning domain: t = 7.08, *p* < 0.001; organization domain: t = 8.05, *p* < 0.001; divided attention domain: t = 6.28, *p* < 0.001). There was also an association between older age, as a covariate, and more severe complaints in the domains of language (t = 2.38, *p* *=* 0*.*018) and organization (t = 2.23, *p* *=* 0*.*027).

When using Aβ status as the measuring method, the results of the ANOVA models showed that there was no main effect of Aβ status on the severity of subjective cognitive complaints after correction in CU individuals (Table 3). This means that CU individuals reported subjective cognitive complaints, in relation to 39 daily activities recruiting different cognitive functions, with similar levels of severity, independently of whether they have high (Aβ+) or low (Aβ−) Aβ levels. However, in MCI individuals, there was a main effect of Aβ status on the severity of three types of memory complaints (“Remembering where I have placed objects”; “Remembering the current date or day of the week”; “Remembering I have already told someone something”), one type of language complaint (“Verbally giving instructions to others”) and one type of planning complaint (“Planning a sequence of stops on a shopping trip”) after correction and after controlling for covariates. This means that MCI individuals who were Aβ+ reported more severe cognitive complaints of these specific types. However, all these effects were small (eta-squared less than 0.09) (see Table 3 for partial eta-squared values). Interestingly, in both CU and MCI individuals, there was a main effect of depressive symptoms, as a covariate, for all types of cognitive complaints, meaning that individuals who reported more depressive symptoms also reported more severe cognitive complaints (see the Supplemental Material). In MCI individuals only, individuals who had higher education, as a covariate, reported less severe complaints about memory (“Repeating stories and/or questions”) [F(1,3969.67, *p* *=* 0*.*026], but more severe complaints about organization (“Keeping living and workspace organized” [F(1397) = 10.33, *p* *=* 0*.*026]; “Keeping mail and papers organized” [F(1398) = 10.98, *p* *=* 0*.*026]), where higher complaint severity typically reflects greater difficulties. No other covariates were significant.

**Table 3.** Two-way (sex X Aβ status) ANOVA results on the severity of 39 types of subjective cognitive complaints from the E-cog questionnaire controlling for age, years of education and symptoms of depression and anxiety.

E-Cog items	CU (F and corrected *p* values)	MCI (F and corrected *p* values)
Sex	Aβ status	Sex X Aβ	Sex	Aβ status	Sex X Aβ
MEMORY 1: Remembering a few shopping items without a list.	F (1,408) = 0.580, *p* *=* 0*.*581	F (1,408) = 0.123, *p* *=* 0*.*943	F (1,408) = 0.018, *p* *=* 0*.*994	F (1,399) = 3.328, *p* *=* 0*.*443	F (1,399) = 5.215, *p* *=* 0*.*112	F (1,399) = 1.229, *p* *=* 0*.*831
MEMORY 2: Remembering things that happened recently (such as recent outings, events in the news).	F (1,408) = 0.079, *p* *=* 0*.*844	F (1,408) = 0.351, *p* *=* 0*.*927	F (1,408) = 0.052, *p* *=* 0*.*969	F (1,400) = 0.450, *p* *=* 0*.*838	F (1,400) = 0.488, *p* *=* 0*.*652	F (1,400) = 0.003, *p* *=* 0*.*993
MEMORY3: Recalling conversations a few days later.	F (1,407) = 4.767, *p* *=* 0*.*163	F (1,407) = 0.009, *p* *=* 0*.*987	F (1,407) = <0.001, *p* *=* 0*.*995	F (1,397) = 0.005, *p* *=* 0*.*945	F (1,397) = 1.749, *p* *=* 0*.*304	F (1,397) = 0.089, *p* *=* 0*.*878
MEMORY4: Remembering where I have placed objects.	F (1,410) = 0.987, *p* *=* 0*.*507	F (1,410) = 0.292, *p* *=* 0*.*927	F (1,410) = 0.058, *p* *=* 0*.*969	F (1,398) = 3.797, *p* *=* 0*.*443	F (1,398) = 11.289, *p* *=* 0*.*011* η^2^_partial_ = 0.02	F (1,398) = 0.125, *p* *=* 0*.*859
MEMORY 5: Repeating stories and/or questions.	F (1,408) = 1.465, *p* *=* 0*.*442	F (1,408) = 0.419, *p* *=* 0*.*927	F (1,408) = 0.380, *p* *=* 0*.*839	F (1,396) = 0.016, *p* *=* 0*.*945	F (1,396) = 4.407, *p* *=* 0*.*158	F (1,396) = 0.953, *p* *=* 0*.*831
MEMORY6: Remembering the current date or day of the week.	F (1,409) = 3.636, *p* *=* 0*.*223	F (1,409) = 2.287, *p* *=* 0*.*782	F (1,409) = <0.001, *p* *=* 0*.*995	F (1,399) = 1.580, *p* *=* 0*.*715	F (1,399) = 18.572, *p* < 0.001*** η^2^_partial_ = 0.04	F (1,399) = 1.279, *p* *=* 0*.*831
MEMORY7: Remembering I have already told someone something.	F (1,406) = 4.744, *p* *=* 0*.*163	F (1,406) = 1.465, *p* *=* 0*.*804	F (1,406) = 2.906, *p* *=* 0*.*539	F (1,395) = 1.343, *p* *=* 0*.*729	F (1,395) = 10.836, *p* *=* 0*.*011* η^2^_partial_ = 0.02	F (1,395) = 2.112, *p* *=* 0*.*831
MEMORY8: Remembering appointments, meetings, or engagements.	F (1,409) = 0.883, *p* *=* 0*.*507	F (1,409) = <0.001, *p* *=* 0*.*987	F (1,409) = 1.330, *p* *=* 0*.*839	F (1,398) = 0.160, *p* *=* 0*.*889	F (1,398) = 4.155, *p* *=* 0*.*164	F (1,398) = 0.122, *p* *=* 0*.*859
LANGUAGE1: Forgetting the names of objects.	F (1,410) = 0.988, *p* *=* 0*.*507	F (1,410) = 3.170, *p* *=* 0*.*782	F (1,410) = 0.429, *p* *=* 0*.*839	F (1,399) = 0.053, *p* *=* 0*.*912	F (1,399) = 1.058, *p* *=* 0*.*424	F (1,399) = 1.017, *p* *=* 0*.*831
LANGUAGE2: Verbally giving instructions to others.	F (1,405) = 3.043, *p* *=* 0*.*240	F (1,405) = 0.057, *p* *=* 0*.*959	F (1,405) = 2.772, *p* *=* 0*.*539	F (1,398) = 0.009, *p* *=* 0*.*945	F (1,398) = 12.178, *p* *=* 0*.*010* η^2^_partial_ = 0.02	F (1,398) = 0.008, *p* *=* 0*.*993
LANGUAGE3: Finding the right words to use in conversations.	F (1,408) = 9.351, *p* *=* 0*.*093	F (1,408) = 0.014, *p* *=* 0.987	F (1,408) = 0.175, *p* *=* 0.910	F (1,399) = 1.001, *p* *=* 0.729	F (1,399) = 0.021, *p* *=* 0.886	F (1,399) < 0.001, *p* *=* 0.993
LANGUAGE4: Communicating thoughts in a conversation.	F (1,408) = 2.323, *p* *=* 0.313	F (1,408) = 0.382, *p* *=* 0.927	F (1,408) = 0.582, *p* *=* 0.839	F (1,399) = 0.190, *p* *=* 0.889	F (1,399) = 1.090, *p* *=* 0.424	F (1,399) = 0.043, *p* *=* 0.931
LANGUAGE5: Following a story in a book or on TV.	F (1,409) = 1.749, *p* *=* 0.383	F (1,409) = 0.855, *p* *=* 0.927	F (1,409) = 0.471, *p* *=* 0.839	F (1,397) = 1.986, *p* *=* 0.715	F (1,397) = 0.251, *p* *=* 0.708	F (1,397) = 0.178, *p* *=* 0.859
LANGUAGE6: Understanding the point of what other people are trying to say.	F (1,408) = 0.497, *p* *=* 0.605	F (1,408) = 1.484, *p* *=* 0.804	F (1,408) = 0.590, *p* *=* 0.839	F (1,398) = 1.899, *p* *=* 0.715	F (1,398) = 0.055, *p* *=* 0.837	F (1,398) = 0.623, *p* *=* 0.857
LANGUAGE7: Remembering the meaning of common words.	F (1,409) = 0.877, *p* *=* 0.507	F (1,409) = 0.348, *p* *=* 0.927	F (1,409) = 1.656, *p* *=* 0.839	F (1,398) = 0.652, *p* *=* 0.780	F (1,398) = 0.327, *p* *=* 0.674	F (1,398) = 0.397, *p* *=* 0.857
LANGUAGE8: Describing a program I have watched on TV.	F (1,402) = 0.203, *p* *=* 0.771	F (1,402) = 2.968, *p* *=* 0.782	F (1,402) = 0.699, *p* *=* 0.839	F (1,388) = 1.037, *p* *=* 0.729	F (1,388) = 2.054, *p* *=* 0.271	F (1,388) = 0.169, *p* *=* 0.859
LANGUAGE9: Understanding spoken directions or instructions.	F (1,410) = 2.962, *p* *=* 0.240	F (1,410) = 0.223, *p* *=* 0.927	F (1,410) = 1.023, *p* *=* 0.839	F (1,399) = 0.019, *p* *=* 0.945	F (1,399) = 1.156, *p* *=* 0.424	F (1,399) = 0.328, *p* *=* 0.857
VISSPAT1: Following a map to find a new location.	F (1,406) = 5.937, *p* *=* 0.135	F (1,406) = 0.004, *p* *=* 0.987	F (1,406) = 2.852, *p* *=* 0.539	F (1,389) = 5.700, *p* *=* 0.443	F (1,389) = 2.103, *p* *=* 0.271	F (1,389) = 0.343, *p* *=* 0.857
VISSPAT2: Reading a map and helping with directions when someone else is driving.	F (1,406) = 3.316, *p* *=* 0.240	F (1,406) = 0.261, *p* *=* 0.927	F (1,406) = 1.152, *p* *=* 0.839	F (1,390) = 4.230, *p* *=* 0.443	F (1,390) = 2.777, *p* *=* 0.221	F (1,390) = 0.155, *p* *=* 0.859
VISSPAT3: Finding my car in a parking lot.	F (1,408) = 4.555, *p* *=* 0.163	F (1,408) = 1.674, *p* *=* 0.804	F (1,408) = 3.705, *p* *=* 0.539	F (1,398) = 3.100, *p* *=* 0.443	F (1,398) = 3.056, *p* *=* 0.210	F (1,398) = 0.196, *p* *=* 0.859
VISSPAT4: Finding my way back to a meeting spot in the mall or other location.	F (1,409) = 0.871, *p* *=* 0.507	F (1,409) = 0.067, *p* *=* 0.959	F (1,409) = 1.234, *p* *=* 0.839	F (1,396) = 0.763, *p* *=* 0.780	F (1,396) = 2.956, *p* *=* 0.210	F (1,396) = 0.644, *p* *=* 0.857
VISSPAT5: Finding my way around a familiar neighborhood.	F (1,410) = 0.091, *p* *=* 0.844	F (1,410) = 0.009, *p* *=* 0.987	F (1,410) = 0.651, *p* *=* 0.839	F (1,399) = 0.141, *p* *=* 0.889	F (1,399) = 2.520, *p* *=* 0.245	F (1,399) < 0.001, *p* *=* 0.993
VISSPAT6: Finding my way around a familiar store.	F (1,410) = 2.114, *p* *=* 0.320	F (1,410) = 2.182, *p* *=* 0.782	F (1,410) = 5.415, *p* *=* 0.399	F (1,399) = 0.115, *p* *=* 0.890	F (1,399) = 2.999, *p* *=* 0.210	F (1,399) = 1.923, *p* *=* 0.831
VISSPAT7: Finding my way around a house visited many times.	F (1,409) = 3.708, *p* *=* 0.223	F (1,409) = 2.186, *p* *=* 0.782	F (1,409) = 0.760, *p* *=* 0.839	F (1,399) = 0.681, *p* *=* 0.780	F (1,399) = 6.569, *p* *=* 0.070	F (1,399) = 0.500, *p* *=* 0.857
PLAN1: Planning a sequence of stops on a shopping trip.	F (1,407) = 5.863, *p* *=* 0.135	F (1,407) = 0.240, *p* *=* 0.927	F (1,407) = 0.395, *p* *=* 0.839	F (1,396) = 0.167, *p* *=* 0.889	F (1,396) = 9.336, *p* *=* **0.019** η^2^_partial_ = 0.02	F (1,396) = 1.563, *p* *=* 0.831
PLAN2: The ability to anticipate weather changes and plan accordingly (i.e., bring a coat or umbrella)	F (1,407) = 6.988, *p* *=* 0.135	F (1,407) = 0.714, *p* *=* 0.927	F (1,407) = 0.056, *p* *=* 0.969	F (1,400) = 1.047, *p* *=* 0.729	F (1,400) = 3.727, *p* *=* 0.176	F (1,400) = 0.832, *p* *=* 0.831
PLAN3: Developing a schedule in advance of anticipated events.	F (1,409) = 3.149, *p* *=* 0.240	F (1,409) = 0.002, *p* *=* 0.987	F (1,409) = 0.096, *p* *=* 0.969	F (1,399) = 3.091, *p* *=* 0.443	F (1,399) = 6.248, *p* *=* 0.071	F (1,399) = 1.431, *p* *=* 0.831
PLAN4: Thinking things through before acting.	F (1,410) = 0.970, *p* *=* 0.507	F (1,410) = 0.284, *p* *=* 0.927	F (1,410) = 0.977, *p* *=* 0.839	F (1,400) = 0.693, *p* *=* 0.780	F (1,400) = 0.323, *p* *=* 0.674	F (1,400) = 2.640, *p* *=* 0.831
PLAN5: Thinking ahead.	F (1,410) = 0.599, *p* *=* 0.581	F (1,410) = 0.160, *p* *=* 0.927	F (1,410) = 0.302, *p* *=* 0.875	F (1,399) = 0.430, *p* *=* 0.838	F (1,399) = 0.326, *p* *=* 0.674	F (1,399) = 2.229, *p* *=* 0.831
ORGAN1: Keeping living and workspace organized.	F (1,409) = 0.379, *p* *=* 0.656	F (1,409) = 0.247, *p* *=* 0.927	F (1,409) = 0.263, *p* *=* 0.879	F (1,397) = 0.363, *p* *=* 0.854	F (1,397) = 1.592, *p* *=* 0.324	F (1,397) = 0.868, *p* *=* 0.831
ORGAN2: Balancing the checkbook without error.	F (1,374) = 2.105, *p* *=* 0.320	F (1,374) = 0.881, *p* *=* 0.927	F (1,374) = 0.001, *p* *=* 0.995	F (1,364) = 0.099, *p* *=* 0.890	F (1,364) = 1.758, *p* *=* 0.304	F (1,364) = 1.141, *p* *=* 0.831
ORGAN3: Keeping financial records organized.	F (1,403) = 0.680, *p* *=* 0.571	F (1,403) = 0.987, *p* *=* 0.927	F (1,403) = 0.418, *p* *=* 0.839	F (1,381) = 1.755, *p* *=* 0.715	F (1,381) = 3.726, *p* *=* 0.176	F (1,381) = 0.321, *p* *=* 0.857
ORGAN4: Prioritizing tasks by importance.	F (1,409) = 5.707, *p* *=* 0.135	F (1,409) = 0.691, *p* *=* 0.927	F (1,409) = 0.189, *p* *=* 0.910	F (1,399) = 0.289, *p* *=* 0.855	F (1,399) = 3.089, *p* *=* 0.210	F (1,399) = 0.500, *p* *=* 0.857
ORGAN5: Keeping mail and papers organized.	F (1,408) = 0.001, *p* *=* 0.994	F (1,408) = 2.244, *p* *=* 0.782	F (1,408) = 0.035, *p* *=* 0.977	F (1,398) = 0.077, *p* *=* 0.897	F (1,398) = 2.272, *p* *=* 0.258	F (1,398) = 3.375, *p* *=* 0.831
ORGAN6: Using an organized strategy to manage a medication schedule involving multiple medications.	F (1,403) = 0.107, *p* *=* 0.844	F (1,403) = 0.071, *p* *=* 0.959	F (1,403) = 0.448, *p* *=* 0.839	F (1,386) = 3.599, *p* *=* 0.443	F (1,386) = 2.335, *p* *=* 0.258	F (1,386) = 1.698, *p* *=* 0.831
DIVATT1: The ability to do two things at once.	F (1,408) < 0.001, *p* *=* 0.994	F (1,408) = 0.164, *p* = 0.927	F (1,403) = 0.651, *p* *=* 0.839	F (1,398) = 0.423, *p* *=* 0.838	F (1,398) = 0.225, *p* *=* 0.708	F (1,398) = 0.993, *p* *=* 0.831
DIVATT2: Returning to a task after being interrupted.	F (1,408) = 1.142, *p* *=* 0.507	F (1,408) = 1.655, *p* *=* 0.804	F (1,408) = 6.037, *p* *=* 0.399	F (1,399) = 1.509, *p* *=* 0.715	F (1,399) = 0.100, *p* *=* 0.792	F (1,399) = 0.386, *p* *=* 0.857
DIVATT3: The ability to concentrate on a task without being distracted by external things in the environment.	F (1,408) = 0.005, *p* *=* 0.993	F (1,408) = 2.389, *p* *=* 0.782	F (1,408) = 3.674, *p* *=* 0.539	F (1,397) = 0.288, *p* *=* 0.855	F (1,397) = 0.348, *p* *=* 0.674	F (1,397) = 0.230, *p* *=* 0.859
DIVATT4: Cooking or working and talking at the same time.	F (1,408) = 2.382, *p* *=* 0.313	F (1,408) = 0.207, *p* *=* 0.927	F (1,408) = 0.005, *p* *=* 0.995	F (1,396) = 1.170, *p* *=* 0.729	F (1,396) = 0.160, *p* *=* 0.747	F (1,396) = 2.249, *p* *=* 0.831

*p < 0.05, **p < 0.01. Significant *p* values are bolded.

Aβ: amyloid-β; CU: cognitively unimpaired; DIVATT: Divided Attention; E-Cog: Everyday Cognition Questionnaire; MCI: mild cognitive impairment; ORGAN: Organization; PLAN: Planification; VISSPAT: Visuospatial

### Objective 2: the severity of subjective cognitive complaints as a predictor of Aβ status, cognitive status and clinical progression

The results of the logistic regressions suggest that, in CU individuals, the severity of none of the 39 subjective cognitive complaints could predict Aβ status (Aβ+/Aβ−), but older age predicted a higher probability of being Aβ+ (z = 2.56, *p* *=* 0.011). It is important to note that this model had a poor fit with the data (McFadden's pseudo-R^2^ = 0.09; R^2^ < 0.1 = weak fit; 0.1 ≤ R^2^ < 0.2 = modest fit; 0.2 ≤ R^2^ < 0.4 = good fit; R^2^ ≥ 0.4 = excellent fit),^72^ so these results should be interpreted with caution. On the other hand, in MCI individuals, a higher probability of being Aβ+ is predicted by more severe complaints in memory (“Remembering where I have placed objects”[z = 2.20, *p* *=* 0.028]), language (“Verbally giving instructions to others” [z = 3.60, *p* < 0.001]) and visuospatial abilities (“Finding my way around a house visited many times” [z = 1.97, *p* *=* 0.049]). Conversely, a *lower* probability of being Aβ+ (or higher probability of being Aβ−) is predicted by more severe, but different, complaints in language (“Forgetting the names of objects” [z = −2.54, *p* *=* 0.011]) and planning (“Thinking ahead” [z = −2.36, *p* *=* 0.018]) domains, suggesting that specific complaint types are differentially useful in detecting increased AD risk. The model ran with MCI individuals had a modest fit with the data (McFadden's pseudo-R^2^ = 0.15)

In addition, the severity of specific subjective cognitive complaints could predict individuals’ cognitive status (CU or MCI) (McFadden's pseudo-R^2^ = 0.22). A higher probability of having MCI was predicted by more severe complaints in memory (“Recalling conversations a few days later” [z = 3.08, *p* *=* 0.002]; “Repeating stories and/or questions” [z = 2.25, *p* *=* 0.024]), planning (“Thinking things through before acting” [z = 2.77, *p* *=* 0.006]) and organization (“Keeping financial records organized” [z = 2.09, *p* *=* 0.036]). Greater symptoms of anxiety also predicted a higher probability of having MCI [z = 2.72, *p* *=* 0.006].

The results of the logistic regressions performed in a complementary analysis reveal that specific complaints can also distinguish progressors (CU to MCI or MCI to AD) from non-progressors (stable CU or MCI). In the CU group (McFadden's pseudo-R^2^ = 0.24), more severe complaints in language (“Understanding spoken directions or instructions” [z = 2.32, *p* *=* 0.021]) and organization (“Keeping financial records organized” [z = 2.06, *p* *=* 0.040]; “Keeping mail and papers organized” [z = 2.09, *p* *=* 0.036]) were associated with a higher likelihood of progressing to MCI. Older age also predicted a higher risk of progression to MCI [z = 2.26, *p* *=* 0.024]. In the MCI group, more severe complaints in language (“Verbally giving instructions to others” [z = 2.11, *p* *=* 0.035]; “Communicating thoughts in a conversation” [z = 2.42, *p* *=* 0.015]) and planning (“Developing a schedule in advance of anticipated events” [z = 2.20, *p* *=* 0.028]) were associated with a higher likelihood of progressing to AD. Conversely, a *lower* probability of AD progression (or higher probability of being MCI stable) is predicted by more severe, but different, complaints in language (“Finding the right words to use in conversations” [z = −2.94, *p* *=* 0.003]) and visuospatial abilities (“Finding my way back to a meeting spot in the mall or other location” [z = −2.02, *p* *=* 0.043]), suggesting that domain-specific complaints are differentially useful in detecting increased risk of clinical progression. The model ran with MCI individuals had a modest fit with the data (McFadden's pseudo-R^2^ = 0.16).

### Objective 3: role of sex

#### Association between Aβ pathology and the severity of subjective cognitive complaints

The result of the general linear models showed that, in CU individuals only, there was an effect of sex only in the visuospatial domain, such that the association between higher Aβ levels and more severe visuospatial complaints was stronger for men than for women, t = −2.01, *p* *=* 0.045 (Figure 1).

#### Severity of cognitive complaints across different domains

The results of the two-way ANOVAs revealed that, both in CU and MCI individuals, the main effect of sex and its interaction with Aβ status on the severity of the 39 different subjective cognitive complaints assessed were not significant after correction and after controlling for covariates (Table 3). This means that all individuals reported subjective cognitive complaints with similar levels of severity independently of their sex, and that the effect of sex did not differ between Aβ+ and Aβ− individuals.

#### Prediction of Aβ status and cognitive status by the severity of subjective cognitive complaints

To determine whether the cognitive complaints that can predict Aβ status (Aβ+/ Aβ−) and cognitive status (CU or MCI) differ between men and women, we performed the same logistic regression models used to address the second objective, but this time separately for men and women.

In CU men, none of the complaints could significantly predict Aβ status, but older age could predict a higher probability of belonging to the Aβ+ group (z = 2.92, *p* *=* 0.004; McFadden's pseudo-R^2^ = 0.25). In CU women, a higher probability of being Aβ+ is predicted by a more severe complaint in organization (“Keeping financial records organized” [z = 2.10, *p* *=* 0.036]). Conversely, a *lower* probability (or higher probability of being Aβ−) is predicted by more severe, but different complaints in language (“Following a story in a book or on TV” [z = −2.16, *p* *=* 0.030]) and divided attention (“Returning to a task after being interrupted” [z = −2.64, *p* *=* 0.008]; “The ability to concentrate on a task without being distracted by external things in the environment” [z = −2.10, *p* *=* 0.036]), where higher complaint severity typically reflects greater difficulties. However, this model only had a modest fit with the data (McFadden's pseudo-R^2^ = 0.19), so these results should be interpreted with caution.

In MCI men, a higher probability of being Aβ+ is predicted by a more severe complaint in language (“Verbally giving instructions to others” [z = 2.86, *p* *=* 0.004]), but this model only had a modest fit with the data (McFadden's pseudo-R^2^ = 0.18), so these results should be interpreted with caution. However, in MCI women, the model had an excellent fit with the data (McFadden's pseudo-R^2^ = 0.55). When controlling for age, the number of years of education and symptoms of depression and anxiety, a higher probability of being Aβ+ is predicted by more severe complaints in memory (“Remembering where I have placed objects”[z = 2.02, *p* *=* 0.043]; “Remembering I have already told someone something” [z = 2.79, *p* *=* 0.005]), language (“Verbally giving instructions to others” [z = 3.18, *p* *=* 0.001]), visuospatial abilities (“Finding my car in a parking lot” [z = 2.40, *p* *=* 0.016]) and organization (“Keeping financial records organized” [z = 2.14, *p* *=* 0.033]). On the other hand, a *lower* probability of being Aβ+ (or higher probability of being Aβ−) is predicted by more severe, but different, complaints in language (“Forgetting the names of objects” [z = −2.46, *p* *=* 0.014]), visuospatial abilities (“Finding my way back to a meeting spot in the mall or other location” [z = −2.52, *p* *=* 0.012]; “Finding my way around a familiar store” [z = −2.63, *p* *=* 0.008]), planning (“Thinking ahead” [z = −2.51, *p* *=* 0.012]) and organization (“Keeping mail and papers organized” [z = −2.87, *p* *=* 0.004]) domains. Interestingly, greater symptoms of anxiety also predicted a higher probability of being Aβ+ in MCI women [z = 2.37, *p* *=* 0.018].

Furthermore, the severity of specific subjective cognitive complaints could predict individuals’ cognitive status (CU or MCI), and these predictors vary based on sex. For men, a higher probability of having MCI was predicted by more severe complaints in memory (“Remembering things that happened recently (such as recent outings, events in the news)” [z = 2.05, *p* *=* 0.040]; “Remembering the current date or day of the week” [z = 2.08, *p* *=* 0.037]) and planning (“Thinking things through before acting” [z = 3.67, *p* < 0.001]), where higher complaint severity typically reflects greater difficulties.Conversely, a *lower* probability (or higher probability of being CU) was predicted by more severe, but different, complaints in visuospatial abilities (“Finding my way around a familiar store” [z = −2.58, *p* *=* 0.010]) and planning (“Thinking ahead” [z = −2.20, *p* *=* 0.028]) (McFadden's pseudo-R^2^ = 0.25). For women, a higher probability of having MCI was also predicted by more severe complaints in memory (“Recalling conversations a few days later” [z = 4.36, *p* < 0.001]; “Remembering appointments, meetings, or engagements” [z = 2.92, *p* *=* 0.003]) and planning (“Planning a sequence of stops on a shopping trip” [z = 1.99, *p* *=* 0.046]), but the complaint types were different from men. Women were also more likely to have MCI if they had greater anxiety symptoms [z = 2.01, *p* *=* 0.044]. Conversely, a *lower* probability of having MCI was predicted by more severe, but different, complaints in memory (“Remembering I have already told someone something” [z = −2.40, *p* *=* 0.017]) and organization (“Balancing the checkbook without error” [z = −2.32, *p* *=* 0.020]) (McFadden's pseudo-R^2^ = 0.33).

## Discussion

Building on prior work linking AD biomarkers and subjective cognitive complaints, our study uniquely integrates complementary aspects of this research area within a single design. Specifically, we examined the relationship between Aβ, using two complementary measures, and the severity of subjective cognitive complaints across different domains, as well as the predictive value of subjective cognitive complaints for Aβ status, cognitive status and clinical progression, leveraging longitudinal data, in CU and MCI individuals. The other innovative contribution of this study was its examination of the role of sex in early AD stages. First, we found an association between higher actual Aβ levels and more severe complaints in language and visuospatial domains in CU individuals only. When using Aβ status, Aβ+ individuals with MCI reported more severe complaints across memory, language, and planning domains compared to Aβ− individuals with MCI. Second, more severe complaints related to memory, language and executive functions (planning and organization) could predict a higher likelihood of MCI and clinical progression from CU to MCI or from MCI to AD. These results show the relevance of assessing subjective cognitive complaints across multiple cognitive domains in early AD stages to identify individuals at heightened risk for pathological progression and cognitive decline. Third, sex differences exist in the association of Aβ levels and visuospatial complaints, with stronger association in CU men than women, highlighting the role of sex in the relationship between AD pathology and the subjective cognitive experience. However, we did not find sex differences in complaints severity across cognitive domains. Thus, despite CU women having higher Aβ levels than men, they did not report more severe complaints, suggesting cognitive resilience in women at early AD stages. Finally, specific types of memory, language, visuospatial and organizational complaints could better predict a higher likelihood of being Aβ+ in women with MCI than in men, and the types of complaints that predict MCI are different between sexes.

Our first objective aimed at exploring the relationship between Aβ pathology and cognitive complaints severity beyond the most frequently investigated domains such as memory and executive functioning in CU and MCI individuals. Our findings suggest an association between higher Aβ levels, measured as continuous SUVR values, and more severe complaints in language and visuospatial domains in CU individuals only. This partially supports our hypothesis, aligning with expectations for the language domain only. These results show the importance of using detailed domain-specific questionnaires like the E-Cog to assess subjective complaints in cognitive domains other than memory to identify individuals at increased risk of AD. While a few studies have found similar associations between Aβ burden (measured in cerebrospinal fluid or via PET) and complaints in language and executive domains,^16,18^ the absence of a relationship with memory complaints contrasts with our initial hypothesis and prior research, which often reports memory complaints as a key early indicator of AD pathology.^12,14,17,19,20^ However, growing evidence also emphasizes the early clinical relevance of language changes, especially in connected speech, as potential markers of preclinical AD.^73,74^ One study even reported that word-finding complaints were among the most common and severe concerns in CU individuals, sometimes exceeding memory-related concerns, and were associated with greater Aβ burden in the brain.^18^ In contrast, Aβ+ MCI individuals reported more severe complaints in the memory, language, and planning (executive function) domains compared to Aβ− MCI individuals. These findings are fully consistent with our hypothesis. While no association with memory complaints was observed in CU individuals, the presence of this link at the MCI stage may reflect a loss of cognitive resilience along the AD continuum. As individuals, particularly women, progress to more advanced stages like MCI, their verbal memory advantage diminishes.^47,48^ As such, their subjective complaints, notably in the memory domain, may become more closely aligned with underlying pathology and cognitive impairment when resilience mechanisms begin to wane.^56,75^ Lastly, the contrasting results between CU and MCI individuals underscore the importance of using both continuous Aβ levels and Aβ+/Aβ− status when studying populations across the AD continuum. In CU individuals, continuous Aβ measures appeared more sensitive, revealing associations with complaints severity that were not detected using binary status. This may be due to the higher proportion of Aβ− individuals in this group, making continuous metrics more effective for capturing subtle pathological changes.^26^ In contrast, among MCI individuals, categorical classification allow for clinically interpretable cutoffs that map to prognosis and are widely used for clinical decisions such as targeting at-risk individuals for intervention,^1,27,28^ clearly distinguishing those with more advanced pathology.

Our second objective aimed to assess which subjective cognitive complaints can predict cerebral Aβ status (Aβ+/Aβ−), cognitive status (CU or MCI), as well as clinical progression (from CU to MCI or from MCI to AD). Our findings showed that none of the complaints significantly predicted Aβ status in CU individuals, contrary to our hypothesis. However, this aligns with the idea that Aβ status classification may lack sensitivity in this population, with subjective cognitive complaints better reflecting Aβ accumulation rather than surpassing a specific threshold. Additionally, as CU individuals typically report *mild* subjective complaints, these may not strongly reflect underlying Aβ burden, further limiting their predictive utility in this group. However, in MCI individuals, the severity of specific types of memory, language and visuospatial complaints could predict Aβ+ status. Moreover, specific complaints related to memory, language and executive functions (planning and organization) could predict a higher probability of MCI and risk of clinical progression (CU to MCI and MCI to AD), which aligns with our hypothesis. These findings further strengthen our previous results, emphasizing the importance of evaluating subjective cognitive complaints beyond the memory domain. Complaints in language and executive functions are not only linked to higher pathology burden but also predict an increased risk of cognitive and functional decline, reinforcing their clinical relevance. Prior work, consistent with our findings, has also linked language and executive function complaints to an increased risk of subsequent cognitive impairment.^8,11,76^ However, most studies did not analyze specific questionnaire items, making our approach more fine-grained and precise. Importantly, whereas criteria for subjective cognitive decline and MCI in many cohorts, including ADNI, often prioritize *memory* complaints, our results highlight the need to assess complaints in other cognitive domains to better capture pathological and clinical progression.

Finally, our third objective aimed at isolating the role of sex in subjective cognitive complaints and AD pathology in early stages. While studying the role of sex in early AD phases has been recognized as a main priority,^77,78^ it is still poorly understood how sex influences the progression and clinical manifestation at the beginning of the disease. Investigating sex differences could then enable more tailored approaches to diagnose and treat AD, potentially improving strategies to slow its progression. Although not directly tested in this study, the theory of cognitive resilience offers a useful framework to interpret our findings on sex differences considering prior research. Cognitive resilience in women has been attributed in part to biological factors, such as the neuroprotective effects of estrogen, which may support neural efficiency and compensate for early AD-related changes.^79^ Additionally, women's lifelong advantage in certain cognitive domains, particularly verbal memory, may allow them to mask or adapt to early cognitive decline, delaying symptom recognition and clinical diagnosis.^56^ This resilience may contribute to a mismatch between pathology and clinical symptoms, since women can tolerate higher pathology burden before showing overt impairment.^80^ First, we found a stronger association between Aβ levels and visuospatial complaints in men compared to women. This supports our hypothesis, suggesting that the weaker association observed in women may reflect cognitive resilience: the idea that, despite similar or even greater levels of Aβ pathology, women are better able to maintain cognitive functioning and might therefore be less likely to report severe complaints that reflect their true pathology load. Previous research also showed that higher cognitive resilience was associated with less subjective cognitive complaints, especially in women.^81,82^ This finding in the visuospatial domain suggests that the impact of Aβ on the subjective experience of decline may be more pronounced in men, despite CU men typically outperforming women on visuospatial tasks,^46^ as also observed in our sample. This suggests that men may experience and report visuospatial decline more directly in relation to Aβ burden, whereas women's greater cognitive reserve may buffer against the subjective impact of similar pathological changes. These results underscore the value of subjective cognitive complaints for studying sex differences in early AD, as sex differences in objective test performance or their association with Aβ pathology may not always be detectable due to ceiling effects or cognitive resilience, whereas subjective perceptions of cognitive change may provide a more sensitive marker for early identification. Interestingly, this effect of sex was not found at theMCI stage. Thus, once women are at a more advanced AD stage, they might lose their cognitive advantage. Indeed, previous studies show that cognitive resilience might delay AD diagnosis or cognitive decline detection in women,^59,62^ but once AD pathology increases at sufficiently high levels, cognitive decline and disease progression is faster and more pronounced for them.^12,47,48^ Although we observed sex differences in the *relationship* between Aβ pathology and specific complaints, we found similar complaints severity between men and women across domains, both in CU and MCI individuals. This finding is inconsistent with our hypothesis, which anticipated that women would report either more or less severe complaints than men, based on prior mixed evidence in the literature. Some previous studies show that women report more frequent and severe complaints than men,^63,65^ others find that men are twice as likely to report complaints,^66,67^ or like us, no difference in complaint prevalence between sexes.^64^ Diverse methodologies to assess subjective cognitive complaints and diverse study populations may explain this inconsistency, restricting our ability to examine sex differences across the full spectrum of AD heterogeneous clinical manifestations. Nevertheless, our result adds to the literature on the role of sex in CU individuals. While we found that CU women had higher Aβ brain levels than CU men, our results suggest that this greater AD pathology burden does not translate into more severe cognitive complaints in daily activities for women. Therefore, complaints may not reliably reflect underlying pathology in women at this stage. This pattern is consistent with the theory of cognitive resilience, which proposes that women are better able to maintain cognitive performance, and possibly the *perception* of intact functioning, despite accumulating AD pathology.^83^ Moreover, we found that specific types of memory, language, visuospatial and organizational complaints better predicted a higher likelihood of being Aβ+ in women with MCI compared to CU women or men. This pattern is consistent with our hypothesis that the predictive value of subjective cognitive complaints may change at different disease stages. This suggests that subjective cognitive complaints may become more accurate indicators of underlying pathology and cognitive impairment in women once they reach more advanced stages of the AD continuum, such as MCI, a point at which their cognitive resilience appears to decline. Finally, while complaints on cognitive domains did not differ between sexes to predict cognitive status, which was contrary to our hypothesis, we found that individual items related to memory and planning differed between men and women in predicting MCI status. Together, these findings highlight the critical importance of integrating sex as a key biological variable in early AD research and clinical evaluation of subjective cognitive complaints. By recognizing that subjective cognitive complaints may carry different meanings and predictive value for men and women at various stages of the disease, clinicians and researchers can move toward more sex-informed diagnostic strategies that improve early detection, risk stratification, and the development of personalized interventions to slow AD progression.

Our study is limited in several ways. First, due to the correlational nature of the research, it is impossible to infer causality regarding the link between Aβ levels and complaints severity. Second, our sample consisted of individuals in the early AD stages, who typically report few or mild cognitive complaints. This can limit the variability in responses on the E-Cog, as most individuals answered using the bottom of the scale (“no change” or “occasionally worse”). While the E-Cog was the only questionnaire available for assessing subjective cognitive complaints in ADNI, future studies could benefit from using questionnaires with a continuous scale that evaluate complaints across cognitive domains. This would allow for a more precise capture of subtle cognitive changes. Our study contributes to support the association between complaints severity and depressive symptoms,^66,68^ highlighting the complexity of subjective cognitive complaints in AD, as they may reflect either mood disorders, which are highly comorbid with AD,^84^ or early AD-related cognitive decline. Notably, depression, which affects more women than men,^85^ especially in older individuals,^86^ is linked to a higher risk of cognitive decline in women.^66^ In our sample, women had greater depressive symptoms than men in the MCI group [t(282) = −2.08, *p* *=* 0.039], but there was no sex difference in CU individuals [t(408) = −1.38, *p* *=* 0.170]. Controlling for affective symptoms such as depression and anxiety is therefore crucial when examining sex differences in complaints. By including these covariates, we ensured that observed sex differences were not confounded by the strong association between depressive symptoms and complaints severity, increasing confidence in our findings. However, severe depressive symptoms are an exclusion criterion in ADNI cohort. Our sample may therefore lack a full range of psychological severity, leading to an underestimation or alteration of the true relationship between depressive symptoms and complaints severity. Finally, anxiety symptoms were measured only with one item of the Neuropsychiatric Inventory. Moreover, ADNI cohort is mainly composed of WEIRD (i.e., Western, Educated, Industrialized, Rich and Democratic)^87^ individuals, which may limit the generalizability of the findings due to the sample's lack of racial and ethnic diversity. Thus, future studies examining subjective cognitive complaints should use a more detailed anxiety questionnaire and a more representative sample, but also investigate gender identity and roles, as these factors may influence the expression of AD symptoms.

In conclusion, our results highlight the importance of investigating subjective cognitive complaints in different domains in early AD stages given the close relationship between Aβ pathology and complaints severity. Subjective cognitive complaints reported by CU and MCI individuals go beyond the memory domain, highlighting the importance to use questionnaires covering a wide range of cognitive domains in the detection of individuals at risk of developing AD and declining cognitively. Our results also suggest to systematically investigate the role of sex in early AD stages because of its influence on the relationship between AD pathology and the expression of cognitive symptoms. A better understanding of sex differences in the AD presentation and progression could enable screening tools to be adapted to better detect individuals at risk despite heterogenous disease profiles.

## Supplemental Material

sj-docx-1-alz-10.1177_13872877251393268 - Supplemental material for Uncovering pathology, subjective cognitive complaints, and sex in early Alzheimer's disease Supplemental material, sj-docx-1-alz-10.1177_13872877251393268 for Uncovering pathology, subjective cognitive complaints, and sex in early Alzheimer's disease by Sophie Boutin, Bérengère Houzé, Alexa Pichet Binette, Simona Maria Brambati and for the Alzheimer's Disease Neuroimaging Initiative in Journal of Alzheimer's Disease
