# Supplementary material for: Uncovering pathology, subjective cognitive complaints, and sex in early Alzheimer's disease
Source: J Alzheimers Dis. 2025 Nov 11;109(1):203–23. doi: 10.1177/13872877251393268 (PMC12722564; doi:10.1177/13872877251393268)
Supplement: sj-docx-1-alz-10.1177_13872877251393268 - Supplemental material for Uncovering pathology, subjective cognitive complaints, and sex in early Alzheimer's disease [file sj-docx-1-alz-10.1177_13872877251393268.docx]

**Supplemental Material**

**Uncovering pathology, subjective cognitive complaints, and sex in early Alzheimer’s disease**

**Supplemental Table 1.** The mean E-Cog subscores and standard deviations per cognitive domain in CU individuals

| **E-Cog score** | **Men_Aβ−**  **(n = 130)** | **Men_Aβ+**  **(n = 55)** | **Women_Aβ−**  **(n = 130)** | **Women_Aβ+**  **(n = 103)** |
| --- | --- | --- | --- | --- |
| Total | 53.07 ± 12.91 | 54.61 ± 17.11 | 53.22 ± 11.92 | 53.36 ± 11.76 |
| Memory | 1.63 ± 0.53 | 1.69 ± 0.63 | 1.67 ± 0.53 | 1.67 ± 0.46 |
| Language | 1.39 ± 0.42 | 1.48 ± 0.54 | 1.46 ± 0.48 | 1.48 ± 0.44 |
| Visuospatial | 1.13 ± 0.25 | 1.23 ± 0.37 | 1.22 ± 0.35 | 1.20 ± 0.31 |
| Planning | 1.21 ± 0.32 | 1.20 ± 0.39 | 1.16 ± 0.28 | 1.16 ± 0.28 |
| Organization | 1.30 ± 0.38 | 1.34 ± 0.49 | 1.29 ± 0.43 | 1.34 ± 0.44 |
| Divided attention | 1.51 ± 0.57 | 1.57 ± 0.60 | 1.63 ± 0.67 | 1.51 ± 0.54 |

Aβ−: amyloid levels below threshold; Aβ+: amyloid levels above threshold; CU: cognitively unimpaired.

**Supplemental Table 2.** The mean E-Cog subscores and standard deviations per cognitive domain in MCI individuals

| **E-Cog score** | **Men_Aβ−**  **(n = 98)** | **Men_Aβ+**  **(n = 150)** | **Women_Aβ−**  **(n = 73)** | **Women_Aβ+**  **(n = 87)** |
| --- | --- | --- | --- | --- |
| Total | 66.89 ± 20.04 | 71.57 ± 22.41 | 67.27 ± 21.00 | 69.33 ± 19.94 |
| Memory | 2.11 ± 0.67 | 2.32 ± 0.71 | 2.15 ± 0.73 | 2.36 ± 0.71 |
| Language | 1.85 ± 0.63 | 1.95 ± 0.66 | 1.87 ± 0.66 | 1.89 ± 0.73 |
| Visuospatial | 1.35 ± 0.47 | 1.51 ± 0.61 | 1.47 ± 0.58 | 1.55 ± 0.58 |
| Planning | 1.43 ± 0.48 | 1.63 ± 0.65 | 1.50 ± 0.61 | 1.48 ± 0.58 |
| Organization | 1.51 ± 0.61 | 1.73 ± 0.73 | 1.65 ± 0.68 | 1.62 ± 0.62 |
| Divided attention | 1.89 ± 0.73 | 1.99 ± 0.76 | 1.93 ± 0.86 | 1.81 ± 0.72 |

Aβ−: amyloid levels below threshold; Aβ+: amyloid levels above threshold; MCI: mild cognitive impairment.

**Supplemental Table 3.** The effect of depressive symptoms, as a covariate in the two-way ANOVA analyses, on the severity of 39 types of subjective cognitive complaints from the E-Cog questionnaire

| **E-Cog items** | **CU (F and corrected *p*)** | **MCI (F and corrected *p*)** |
| --- | --- | --- |
| MEMORY 1:  Remembering a few shopping items without a list. | F (1,408) = 40.764  *p* < **0.001***** | F (1,399) = 30.329  *p* < **0.001***** |
| MEMORY 2:  Remembering things that happened recently (such as recent outings, events in the news). | F (1,408) = 15.396  *p* < **0.001***** | F (1,400) = 19.741  *p* < **0.001***** |
| MEMORY3:  Recalling conversations a few days later. | F (1,407) = 33.396  *p* < **0.001***** | F (1,397) = 26.723  *p* < **0.001***** |
| MEMORY4:  Remembering where I have placed objects. | F (1,410) = 20.917  *p* < **0.001***** | F (1,398) = 26.434  *p* < **0.001***** |
| MEMORY 5:  Repeating stories and/or questions. | F (1,408) = 21.620  *p* < **0.001***** | F (1,396) = 16.462  *p* < **0.001***** |
| MEMORY6:  Remembering the current date or day of the week. | F (1,409) = 8.343  *p =* **0*.*004**** | F (1,399) = 38.407  *p* < **0.001***** |
| MEMORY7:  Remembering I have already told someone something. | F (1,406) = 12.497  *p* < **0.001***** | F (1,395) = 15.711  *p* < **0.001***** |
| MEMORY8:  Remembering appointments, meetings, or engagements. | F (1,409) = 40.742  *p* < **0.001***** | F (1,398) = 42.837  *p* < **0.001***** |
| LANGUAGE1:  Forgetting the names of objects. | F (1,410) = 17.373  *p* < **0.001***** | F (1,399) = 25.709  *p* < **0.001***** |
| LANGUAGE2:  Verbally giving instructions to others. | F (1,405) = 35.253  *p* < **0.001***** | F (1,398) = 37.686  *p* < **0.001***** |
| LANGUAGE3:  Finding the right words to use in conversations. | F (1,408) = 45.047  *p* < **0.001***** | F (1,399) = 29.838  *p* < **0.001***** |
| LANGUAGE4:  Communicating thoughts in a conversation. | F (1,408) = 62.474  *p* < **0.001***** | F (1,399) = 32.862  *p* < **0.001***** |
| LANGUAGE5:  Following a story in a book or on TV. | F (1,409) = 15.732  *p* < **0.001***** | F (1,397) = 43.518  *p* < **0.001***** |
| LANGUAGE6:  Understanding the point of what other people are trying to say. | F (1,408) = 10.793  *p* < **0.001***** | F (1,398) = 31.294  *p* < **0.001***** |
| LANGUAGE7:  Remembering the meaning of common words. | F (1,409) = 12.928  *p* < **0.001***** | F (1,398) = 16.578  *p* < **0.001***** |
| LANGUAGE8:  Describing a program I have watched on TV. | F (1,402) = 12.300  *p* < **0.001***** | F (1,388) = 30.047  *p* < **0.001***** |
| LANGUAGE9:  Understanding spoken directions or instructions. | F (1,410) = 15.069  *p* < **0.001***** | F (1,399) = 18.451  *p* < **0.001***** |
| VISSPAT1:  Following a map to find a new location. | F (1,406) = 6.922  *p =* **0*.*009**** | F (1,389) = 30.950  *p* < **0.001***** |
| VISSPAT2:  Reading a map and helping with directions when someone else is driving. | F (1,406) = 16.896  *p* < **0.001***** | F (1,390) = 39.045  *p* < **0.001***** |
| VISSPAT3:  Finding my car in a parking lot. | F (1,408) = 8.004  *p =* **0*.*005**** | F (1,398) = 35.972  *p* < **0.001***** |
| VISSPAT4:  Finding my way back to a meeting spot in the mall or other location. | F (1,409) = 16.623  *p* < **0.001***** | F (1,396) = 38.700  *p* < **0.001***** |
| VISSPAT5:  Finding my way around a familiar neighborhood. | F (1,410) = 26.991  *p* < **0.001***** | F (1,399) = 25.671  *p* < **0.001***** |
| VISSPAT6:  Finding my way around a familiar store. | F (1,410) = 25.751  *p* < **0.001***** | F (1,399) = 26.135  *p* < **0.001***** |
| VISSPAT7:  Finding my way around a house visited many times. | F (1,409) = 9.249  *p =* **0*.*003**** | F (1,399) = 15.363  *p* < **0.001***** |
| PLAN1:  Planning a sequence of stops on a shopping trip. | F (1,407) = 26.586  *p* < **0.001***** | F (1,396) = 22.984  *p* < **0.001***** |
| PLAN2:  The ability to anticipate weather changes and plan accordingly (i.e., bring a coat or umbrella) | F (1,407) = 4.017  *p =* **0*.*046*** | F (1,400) = 22.448  *p* < **0.001***** |
| PLAN3:  Developing a schedule in advance of anticipated events. | F (1,409) = 27.480  *p* < **0.001***** | F (1,399) = 44.727  *p* < **0.001***** |
| PLAN4:  Thinking things through before acting. | F (1,410) = 30.572  *p* < **0.001***** | F (1,400) = 39.345  *p* < **0.001***** |
| PLAN5:  Thinking ahead. | F (1,410) = 42.651  *p* < **0.001***** | F (1,399) = 41.064  *p* < **0.001***** |
| ORGAN1:  Keeping living and workspace organized. | F (1,409) = 21.187  *p* < **0.001***** | F (1,397) = 37.855  *p* < **0.001***** |
| ORGAN2: Balancing the checkbook without error. | F (1,374) = 13.229  *p* < **0.001***** | F (1,364) = 23.674  *p* < **0.001***** |
| ORGAN3:  Keeping financial records organized. | F (1,403) = 26.978  *p* < **0.001***** | F (1,381) = 36.693  *p* < **0.001***** |
| ORGAN4:  Prioritizing tasks by importance. | F (1,409) = 34.625  *p* < **0.001***** | F (1,399) = 41.970  *p* < **0.001***** |
| ORGAN5:  Keeping mail and papers organized. | F (1,408) = 26.568  *p* < **0.001***** | F (1,398) = 54.104  *p* < **0.001***** |
| ORGAN6:  Using an organized strategy to manage a medication schedule involving multiple medications. | F (1,403) = 15.054  *p* < **0.001***** | F (1,386) = 40.506  *p* < **0.001***** |
| DIVATT1:  The ability to do two things at once. | F (1,408) = 18.822  *p* < **0.001***** | F (1,398) = 24.796  *p* < **0.001***** |
| DIVATT2:  Returning to a task after being interrupted. | F (1,408) = 30.310  *p* < **0.001***** | F (1,399) = 39.237  *p* < **0.001***** |
| DIVATT3:  The ability to concentrate on a task without being distracted by external things in the environment. | F (1,408) = 25.866  *p* < **0.001***** | F (1,397) = 31.677  *p* < **0.001***** |
| DIVATT4:  Cooking or working and talking at the same time. | F (1,408) = 30.769  *p* < **0.001***** | F (1,396) = 23.375  *p* < **0.001***** |

**p*<0.05, ***p*<0.01, ****p*<0.001

Aβ: amyloid β; CU: cognitively unimpaired; DIVATT: Divided Attention; E-Cog: Everyday Cognition Questionnaire; MCI: mild cognitive impairment; ORGAN: Organization; PLAN: Planification; VISSPAT: Visuospatial

**Supplemental Figure 1.** The association between Aβ levels and the severity of cognitive complaints, by domain, controlling for age, years of education and symptoms of depression and anxiety in CU men.

*p =* 0*.*836

*p =* 0*.*292

*p =* **0*.*034***

*p =* 0*.*880

*p =* 0*.*345

*p =* 0*.*060

*p =* 0*.*227

**p*<0.05. Reported p-values reflect the associations between Aβ levels and E-Cog scores. Higher amyloid β levels were associated with higher E-Cog scores (more severe subjective cognitive complaints) only in the visuospatial domain in CU men.

CU: Cognitively Unimpaired; E-Cog: Everyday Cognition Questionnaire; SUVR: Standardized Uptake Value Ratio.

**Supplemental Figure 2.** The association between Aβ levels and the severity of cognitive complaints, by domain, controlling for age, years of education and symptoms of depression and anxiety in CU women.

*p =* **0*.*040***

*p =* 0*.*916

*p =* 0*.*640

*p =* 0*.*336

*p =* 0*.*706

*p =* 0*.*362

*p =* 0*.*815

**p*<0.05. Reported p-values reflect the associations between Aβ levels and E-Cog scores. Higher amyloid β levels were associated with higher E-Cog scores (more severe subjective cognitive complaints) only in the organization domain in CU women.

CU: Cognitively Unimpaired; E-Cog: Everyday Cognition Questionnaire; SUVR: Standardized Uptake Value Ratio.

**Supplemental Figure 3.** The association between Aβ levels and the severity of cognitive complaints, by domain, controlling for age, years of education and symptoms of depression and anxiety in MCI men.

*p =* 0*.*132

*p =* 0*.*803

*p =* 0*.*124

*p =* 0*.*319

*p =* 0*.*179

*p =* 0*.*252

*p =* 0*.*412

Reported p-values reflect the associations between Aβ levels and E-Cog scores. Amyloid β levels were not associated with E-Cog scores in any cognitive domain in MCI men.

E-Cog: Everyday Cognition Questionnaire; MCI: Mild Cognitive Impairment; SUVR:, Standardized Uptake Value Ratio.

**Supplemental Figure 4.** The association between Aβ levels and the severity of cognitive complaints, by domain, controlling for age, years of education and symptoms of depression and anxiety in MCI women

*p =* 0*.*371

*p =* 0*.*438

*p =* 0*.*994

*p =* 0*.*656

*p =* 0*.*353

*p =* **0*.*017***

*p =* 0*.*385

**p*<0.05. Reported p-values reflect the associations between Aβ levels and E-Cog scores. Higher amyloid β levels were associated with higher E-Cog scores (more severe subjective cognitive complaints) only in the memory domain in MCI women.

E-Cog: Everyday Cognition Questionnaire; MCI: Mild Cognitive Impairment; SUVR: Standardized Uptake Value Ratio.
